# Recombinant production of human α_2_-macroglobulin variants and interaction studies with recombinant G-related α_2_-macroglobulin binding protein and latent transforming growth factor-β_2_

**DOI:** 10.1038/s41598-019-45712-z

**Published:** 2019-06-24

**Authors:** Laura Marino-Puertas, Laura del Amo-Maestro, Marta Taulés, F. Xavier Gomis-Rüth, Theodoros Goulas

**Affiliations:** 1 Proteolysis Laboratory, Structural Biology Unit (“Maria de Maeztu” Unit of Excellence), Molecular Biology Institute of Barcelona, Higher Scientific Research Council (CSIC), Barcelona Science Park, Helix Building; Baldiri Reixac, 15-21, 08028 Barcelona, Catalonia Spain; 2 Scientific and Technological Centers (CCiTUB), University of Barcelona, Lluís Solé i Sabaris, 1-3, 08028 Barcelona, Catalonia Spain

**Keywords:** Biochemistry, Biological techniques

## Abstract

α_2_-Macroglobulins (α_2_Ms) regulate peptidases, hormones and cytokines. Mediated by peptidase cleavage, they transit between native, intact forms and activated, induced forms. α_2_Ms have been studied over decades using authentic material from primary sources, which was limited by sample heterogeneity and contaminants. Here, we developed high-yield expression systems based on transient transfection in *Drosophila* Schneider 2 and human Expi293F cells, which produced pure human α_2_M (hα_2_M) at ~1.0 and ~0.4 mg per liter of cell culture, respectively. In both cases, hα_2_M was mainly found in the induced form. Shorter hα_2_M variants encompassing N-/C-terminal parts were also expressed and yielded pure material at ~1.6/~1.3 and ~3.2/~4.6 mg per liter of insect or mammalian cell culture, respectively. We then analyzed the binding of recombinant and authentic hα_2_M to recombinant latent human transforming growth factor-β_2_ (pro-TGF-β_2_) and bacterial G-related α_2_M binding protein (GRAB) by surface plasmon resonance, multiple-angle laser light scattering, size-exclusion chromatography, fluorogenic labelling, gel electrophoresis and Western-blot analysis. Two GRAB molecules formed stable complexes of high affinity with native and induced authentic hα_2_M tetramers. The shorter recombinant hα_2_M variants interacted after preincubation only. In contrast, pro-TGF-β_2_ did not interact, probably owing to hindrance by the N-terminal latency-associated protein of the cytokine.

## Introduction

α_2_-Macroglobulins (α_2_Ms) are large protein inhibitors, which counteract a broad spectrum of endopeptidases. To date, they have been characterized from metazoans and Gram-negative bacteria^1–4^. They are multi-domain molecular traps with comparable structural and biochemical properties, which present related modes of action termed “Venus flytrap” and “snap-trap” mechanisms^5,6^. In both cases, peptidases cut native α_2_M in a highly flexible bait region, which triggers a massive conformational rearrangement that induces the inhibitor and entraps the peptidase. In some family members, a second event involves a highly reactive β-cysteinyl-γ-glutaminyl thioester bond, which is activated by nucleophiles such as lysines and covalently binds the prey peptidase, thus contributing to the stabilization of the enzyme:inhibitor complex. Trapped peptidases are still active but only against small substrates due to steric hindrance^7^. Hence, α_2_Ms regulate proteolysis in complex biological processes such as digestion, blood homeostasis, signaling, tissue remodeling and defense against toxins and other virulence factors during infection and envenomation^1^.

**Figure 1:**
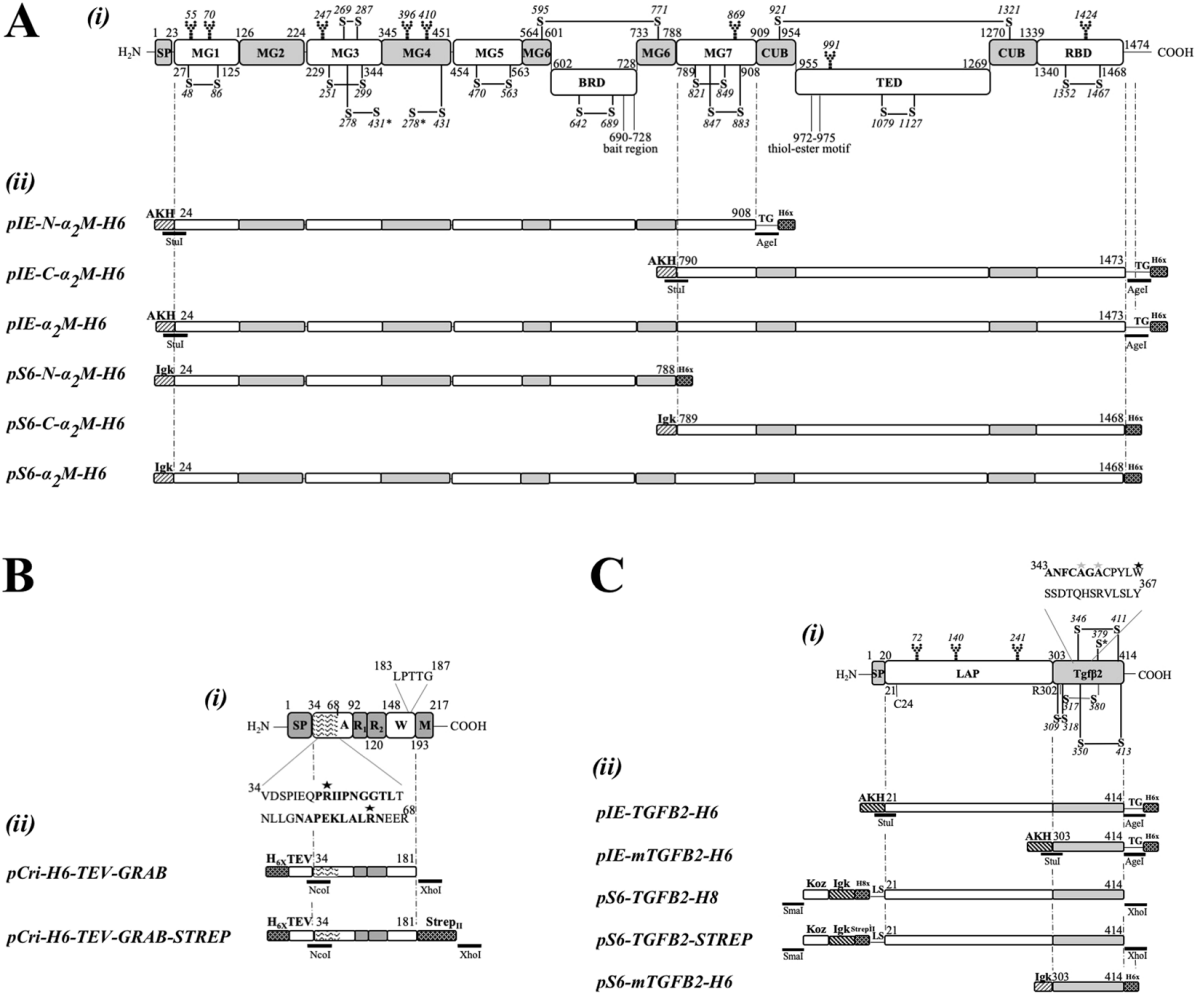
Overview of studied proteins. (**A**) Scheme depicting the domain structure of hα_2_M (*i*) and the constructs studied (*ii*). The residue numbers correspond to UP P01023. (*i*) Functional regions and domains are the signal peptide (SP); macroglobulin domains 1-to-7 (MG1-MG7); the bait-region domain (BRD); the CUB domain; the thioester domain (TED); and the receptor-binding domain (RBD). Disulfide bonds are shown in black and linked cysteines are labelled. An interchain disulfide is pinpointed with an asterisk and *N*-linked glycosylation sites are highlighted with a sugar chain. (*ii*) hα_2_M fusion proteins produced with plasmids pIEx and pCMV-Sport6. The AKH signal peptide sequence, the mouse Ig κ-chain leader sequence, His_6×_-tags and restriction sites are graphically represented. **(B)** Same as (A) for GRAB (UP Q7DAL7). (*i*) Functional regions and domains are the SP; domain A, with the binding regions of hα_2_M hatched and in the inset; repeat regions R_1_ and R_2_; the cell-wall attachment site (W), with the cell-wall anchor motif shown in magnification; and the membrane anchor (M). Critical arginine residues for hα_2_M-binding are indicated by a star (R^42^ and R^64^). (*ii*) GRAB fusion proteins in pCri8a with His_6_-tag, TEV site and Strep-tag. **(C)** Same as (A) for pro-TGF-β_2_ (UP P61812). (*i*) Functional domains and regions are the SP; the latency associated peptide (LAP); and the mature growth factor moiety (TGF-β_2_). Critical residues in LAP are C^24^, which is involved in the binding of LTBP, and R^302^, required for furin cleavage. Mature TGF-β_2_ segment A^343^-Y^367^ is involved in hα2M binding, important and critical residues are indicated by a grey (A^347^ and A^349^) and a black star (W^354^), respectively. (*ii*) Human pro-TGF-β_2_ fusion proteins produced with plasmids pIEx and pCMV-Sport6. The AKH signal peptide sequence, the Kozac (Koz), the mouse Igκ-chain leader sequence, affinity tags (His_6_ and Strep) and restriction sites are graphically represented.

In addition to peptidase binding and inhibition, α_2_Ms regulate several other endogenous and exogenous proteins (for a complete list, see^1^ and refences therein). Indeed, eukaryotic α_2_Ms modify and modulate the activity of cytokines, hormones, growth factors, lipid factors and other proteins, and thus have a great impact on human physiology. A characteristic example is the interaction of human α_2_M (hα_2_M), a 1,474-residue tetrameric multidomain protein (Fig. 1A), with transforming growth factors-β (TGF-βs), a family of ~25-kDa structurally homologous dimeric proteins (Fig. 1C). In mammals, the TGF-β family has three members (TGF-β_1_, TGF-β_2_ and TGF-β_3_), which share 70% sequence identity and similar three-dimensional structures^8^. Their biological activity includes growth regulation, transcriptional activation of extracellular-matrix-related genes and chemotactic activity^9,10^. They are primarily regulated by the non-covalently attached N-terminal latency-associated domain (LAP)^11^, which acts as a pro-domain in the latent ~100-kDa pro-forms (pro-TGF-βs). Once in circulation, LAP is removed and TGF-β availability is regulated by hα_2_M, which sequesters most of these cytokines through a currently unknown mechanism^10,12,13^. What is known is that hα_2_M positions E^753^, E^737^ and D^742^ within segment V^723^-T^761^ (numbering according to UniProt [UP] entry P01023) are involved in TGF-β_1_ binding^14^ and that induced hα_2_M binds the cytokine with higher affinity than the native inhibitor^14^.

The functional and structural properties of hα_2_M are exploited by pathogens such as *Streptococcus pyogenes* (group A streptococci), which forms stable interactions with hα_2_M by a surface protein, the G-related α_2_M-binding protein (GRAB^15,16^). This 23-kDa protein consists of a Gram-positive membrane anchor motif, a variable number of 28-residue repeats, and a highly-conserved N-terminal domain responsible for the interaction with hα_2_M (Fig. 1B). By recruiting native hα_2_M to the membrane, GRAB provides *S*. *pyogenes* with a mechanism to inhibit host peptidases, which protects bacterial surface structures and facilitates progressive dissemination in the infected tissue^15^.

These interactions have only been preliminary characterized^17,18^, and the mechanisms are still unknown. To shed light on them, we developed eukaryotic expression systems of hα_2_M variants and purified the authentic protein from blood. We further used these proteins to study complex formation with GRAB and pro-TGF-β_2_ by several biophysical approaches.

## Materials and Methods

### Construct preparation

Constructs spanning fragments of the gene coding for hα_2_M, namely full-length hα_2_M and its N- and C-terminal parts (N-hα_2_M and C-hα_2_M; for details on constructs, plasmids, vectors and primers, see Table 1 and Fig. 1), and the coding sequence for GRAB from *Streptococcus pyogenes* serotype M1 (UP Q7DAL7) were amplified with primers that introduced either restriction sites for directional cloning or overhangs for restriction-free cloning. The vectors used were pCri-8a^19^ for bacterial expression, pIEx (Novagen) for expression in *Drosophila melanogaster* Schneider 2 embryonic cells (S2; Gibco), and pCMV-Sport 6 (Thermo Scientific) for expression in human Expi293F^™^ cells (Gibco). Polymerase chain reaction (PCR) primers and DNA modifying enzymes were purchased from Sigma-Aldrich and Thermo Scientific, respectively. PCR was performed using Phusion High Fidelity DNA polymerase (Thermo Scientific) according to the manufacturer’s instructions and following a standard optimization step by thermal gradient in each reaction. Mutants were generated by a modified version of the previously described procedure^20^. DNA was purified with the OMEGA Biotek Purification Kit according to the manufacturer’s instructions, and all constructs were verified by DNA sequencing.

**Table 1:** Constructs, primers, plasmids and proteins.

Plasmid name	Protein	Parental DNA	Forward -primer*	Reverse-primer*	Protein sequence**	Tags***	Comments
*pIE-hα*_2_ *M-H6*	hα_2_M	Human c-DNA pIEx vector	CATTAGGCCTCAGTCTCTGGAAAACCGCAGTATATG	CATTACCGGTAGGATTTCCAAGATCTTTG	S^24^-N^1473^ + **PTG** + **H**_**6×**_	C-t H_6×_	Full-length hα_2_M in S2 cells. The gene was inserted by directional cloning (between *Stu*I and *Age*I) into the pIEx vector in frame with the AKH signal peptide sequence.
*pIE-N-hα*_2_ *M-H6*	N-hα_2_M	pIE-hα_2_M-H6	GATCTTGGAAATCCTACCGGTCATCATCAC	CAATACCGGTTTCAGGTTCAACCAACAGAG	S^24^-E^908^ + **TG** + **H**_**6×**_	C-t H_6×_	As above for the N-terminal half of hα_2_M.
*pIE-C-hα*_2_ *M-H6*	C-hα_2_M	pIE-hα_2_M-H6	CAATAGGCCTCACAGCCCTTCTTTGTGGAGCTC	CAATAGGCCTCAGCGATGATGACGAAAG	Q^790^- N^1473^ + **TG** + **H**_**6×**_	C-t H_6×_	As above for the C-terminal half of hα_2_M.
*pS6-hα*_2_ *M-H6*	hα_2_M	pIE-hα_2_M-H6 pCMV-Sport 6 vector	TGGGTTCCAGGTTCCACTGGTGACTCAGTCTCTGGAAAACCGCAGTAT	CGCCTAATGGTGATGGTGATGGTGGCTGCAAGGAGCATTGTACTCAGC	S^24^-S^1468^ + **H**_**6×**_	C-t H_6×_	Full-length hα_2_M in Expi293F cells. The gene was inserted by restriction-free cloning into the pCMV-Sport 6 vector in frame with the Ig κ leader sequence.
*pS6-N-hα*_2_ *M-H6*	N-hα_2_M	pIE-N-hα_2_M-H6 pCMV-Sport 6 vector	TGGGTTCCAGGTTCCACTGGTGACTCAGTCTCTGGAAAACCGCAGTAT	CGCCTAATGGTGATGGTGATGGTGGGCTCGGAGAGAGGCAGTGGAAGA	S^24^-A^788^ + **H**_**6×**_	C-t H_6×_	As above for the N-terminal half of hα_2_M.
*pS6-C-hα*_2_ *M-H6*	C-hα_2_M	pIE-hα_2_M-H6 pCMV-Sport 6 vector	TGGGTTCCAGGTTCCACTGGTGACTTCCAGCCCTTCTTTGTGGAGCTC	CGCCTAATGGTGATGGTGATGGTGGCTGCAAGGAGCATTGTACTCAGC	F^789^-S^1468^ + **H**_**6×**_	C-t H_6×_	As above for the C-terminal half of hα_2_M.
*pCri-H6-TEV-GRAB*	GRAB	Synthetic DNA pCri8a vector	CAATCCATGGTTGATAGCCCGATTG	CAATCTCGAGTTAATTAACGTTCTGACGTT	**GAM** + V^34^-N^181^	N-t H_6× _+TEV	Synthetic gene of GRAB optimized for expression in *Escherichia coli* inserted into the pCri8a vector^19^ by directional cloning between the *Nco*I and *Xho*I restriction sites.
*pCri-H6-TEV-GRAB-STREP*	GRAB	pCri-H6-TEV-GRAB	ATGCCCATGGTTGATAGCCCG	CGAATTGTGGATGGCTCCAACCTCCATTAACGTTCTGACGTTC; CTTCCACCTCCAGAACCTCCACCCTTTTCGAATTGTGGATGGCTCC; GTGGATGGCTCCATGCGCTACCTCCACTTCCACCTCCAGAACC; GCATCTCGAGTTACTTTTCGAATTGTGGATGGCTCCATGCGC	**GAM **+ V^34^-N^181^ + **GGWSHPQFEKGGGSGGGSGGSAWSHPQFEK**	N-t H_6× _+TEV-(protein)-Strep	This construct was obtained from pCri-H6-TEV-GRAB by four consecutive PCR reactions to introduce a C-terminal Strep-tag.
*pIE-TGFB2-H6*	pro-TGF-β_2_	Human c-DNA pIEx vector	CAATAGGCCTTGTCTACCTGCAGCACACTC	CAATACCGGTGCTGCATTTGCAAGACTTTAC	L^21^-S^414^ + **TG** + **H**_**6×**_	C-t H_6×_	Pro-TGF-β_2_ in S2 cells. The gene was inserted by directional cloning (between *Stu*I and *Age*I) into the pIEx vector in frame with AKH signal peptide sequence
*pIE-mTGFB2-H6*	TGF-β_2_	Human c-DNA pIEx vector	CAATAGGCCTCAGCTTTGGATGCGGCCTATTG	CAATACCGGTGCTGCATTTGCAAGACTTTAC	A^303^-S^414^ + **TG** + **H**_**6×**_	C-t H_6×_	As above for mature TGF-β_2_.
*pS6-TGFB2-H8*	pro-TGF-β_2_	pIE-TGFB2-H6 pCMV-Sport 6 vector	TCACCACCACCATCATCTCAGCCTGTCTACCTGCAGCA; GGTTCCACTGGTGACCACCACCATCACCACCACCATC; GGGTACTGCTGCTCTGGGTTCCAGGTTCCACTGGTGAC; GACAGACACACTCCTGCTATGGGTACTGCTGCTC; CAATCCCGGGGCCACCATGGAGACAGACACACTCC	CAATCTCGAGCTAGCTGCATTTGCAAGACTTTAC	**H**_**8×**_ + **LS** + L^21^-S^414^	N-t H_8×_	Pro-TGF-β_2_ in Expi293F cells. See^8^ for details.
*pS6-TGFB2-STREP*	pro-TGF-β_2_	pS6-TGFB2-H8	GGTGGAGGTTCTGGAGGTGGAAGTGGAGGTAGCGCATGGAGCCATCCACAATTCGAAAAGCTCAGCCTGTCTACCTGC	CTTTTCGAATTGTGGATGGCTCCAGTCACCAGTGGAACCTGGAACCCAGAGCAG	**WSHPQFEKGGGSGGGSGGSAWSHPQFEKLS** + L^21^-S^414^	N-t Strep	Pro-TGF-β_2_ in Expi293F cells. The parental plasmid was modified by opposite primers to replace the N-terminal histidine-tag with a Strep-tag. See^8^ for details.
*pS6-mTGFB2-H6*	TGF-β_2_	pIE-mTGFB2-H6 pCMV-Sport 6 vector	GTTCCAGGTTCCACTGGTGACGCTTTGGATGCGGCCTATTGC	CCTAATGGTGATGGTGATGGTGGCTGCATTTGCAAGACTTTACA	A^303^-S^414^ + **H**_**6×**_	C-t H_6×_	Mature TGF-β_2_ in Expi293F cells. The coding gene extracted from the parental plasmid was inserted into the pCMV-Sport 6 vector by restriction-free cloning between the Ig κ leader sequence and the C-terminal histidine-tag.

All constructs are for extracellular expression of the respective proteins.

*Restriction-site sequences and overhangs for restriction-free cloning are underlined.

**Peptide sequence of the expressed protein after fusion-tag removal. Amino acids derived from the construct are in bold. See also Fig. 1.

***Fused tags at the carboxy-terminus (C-t) or the amino-terminus (N-t).

AKH, adipokinetic hormone; TEV, tobacco-etch virus peptidase; Ig κ, immunoglobulin κ.

### Cell-culture media

S2 and Expi293F cells were adapted to grow in suspension in Sf-900^™^ II SFM culture medium (Gibco) and FreeStyle^™^ F17 expression medium (Gibco) with 0.2% Pluronic F-68 (Gibco) *plus* 8 mM L-glutamine (Gibco), respectively. Both growth media were supplemented with 0.5 µg/mL of the antimycotic Fungizone, 100 units/mL of penicillin, and 100 µg/mL of streptomycin sulfate (Gibco).

### Cell-culture growth

S2 cells were cultivated in TubeSpin bioreactor tubes (TS50 for 5-to-10-mL cultures and TS600 for 100-to-200-mL cultures; Techno Plastic Products AG) as previously described^21^. Cells were passaged three times per week to a final density of 4 × 10^6^ cells/mL. The cultures were incubated at 28 °C in a shaker (Brunswick Scientific Innova) under agitation at 220 rpm.

Expi293F cells were cultivated in 125-mL or 1000-mL polycarbonate Erlenmeyer flasks (FPC0125S and FPC1000S, respectively; Tri Forest Labware) for 25-to-30-mL and 100-to-250-mL cultures, respectively. Cells were subcultured three times per week to a final density of 0.3–0.5 × 10^6^ cells/mL and kept in suspension at 150 rpm in a Multitron Cell Shaker Incubator (Infors HT) at 37 °C in a modified atmosphere (8% CO_2_ and 85% of relative humidity). Cell densities and viability were determined by the trypan blue exclusion test^22^.

### Cell-culture transfection

Linear 25-kDa polyethylenimine (PEI; Polysciences Europe GmbH) was prepared in Milli-Q water at a concentration of 1 mg/mL and pH 7.0. The solution was filter-sterilized and stored at −20 °C. Plasmid DNA was produced in *Escherichia coli* DH5α cells, purified with the GeneJET Plasmid Maxiprep Kit (Thermo Scientific), and stored at −20 °C in sterile Milli-Q water at 1 mg/mL.

For transfection, S2 cells were centrifuged and resuspended in prewarmed fresh medium to a cell density of 15 × 10^6^ cells/mL. A mixture of 0.6 μg DNA (see Fig. 1 and Tables 1) and 2 μg PEI per 1 × 10^6^ cells and per prewarmed transfection volume was pre-incubated for 15–20 min at room temperature and then added dropwise to the cell cultures. These were further incubated for 1 hour at 28 °C and 220 rpm, subsequently diluted with prewarmed fresh medium to 5 × 10^6^ cells/ml and harvested after seven days for protein purification.

For mammalian cultures, Expi293F cells were transfected at a cell density of 1 × 10^6^ cells/mL with a mixture of 1 mg of DNA (see Table 1) and 3 mg of PEI in 20 mL of Opti-MEM Medium (Gibco) per liter of expression medium. The DNA-PEI mixture was incubated at room temperature for 15–20 min and then added dropwise to the cell cultures, which were harvested after three days for protein purification.

### Bacterial expression

For the recombinant overexpression of N-terminally hexa-histidine (His_6_)-tagged GRAB with a tobacco-etch virus peptidase (TEV) recognition sequence, with or without an additional C-terminal Streptactin ®II tag (Strep-tag; IBA Life Sciences), plasmid pCRI8a^19^ was transformed into *E*. *coli* BL21 (DE3) cells (Novagen^23^), and cultures were grown in lysogeny broth supplemented with 30 μg/mL kanamycin. After initial growth at 37 °C to an *OD*_600_ ≈ 0.6, cultures were cooled to 20 °C, and protein expression was induced with 0.4 mM isopropyl-β-D-thiogalactopyranoside for 18–20 hours.

### Protein purification

Protein purification steps were performed at 4 °C if not otherwise stated. For GRAB purification, bacterial cells were collected by centrifugation at 6,000 × *g* for 30 min, washed in buffer A (50 mM Tris-HCl, 250 mM sodium chloride, pH 7.5) and resuspended in the same buffer *plus* 20 mM imidazole, Complete EDTA-free Peptidase Inhibitor Cocktail Tablets and DNase I (both from Roche Diagnostics). Cells were lyzed with a cell disrupter (Constant Systems) at a pressure of 1.35 kbar, cell debris was removed by centrifugation at 30,000 × *g* for 1 hour, and the supernatant containing GRAB was kept for subsequent purification steps. For the hα_2_M variants produced in S2 and Expi293F systems, cells were removed by centrifugation at 2,800 × *g* for 20 min and the supernatant was used for subsequent purification steps.

Supernatants containing the proteins of interest were incubated for 20 min (expression in insect cells) or 1 hour (expression in mammalian cells) with nickel-nitrilotriacetic acid resin (Ni-NTA; Invitrogen), which was subsequently loaded onto an open column for batch purification (Bio-Rad), washed extensively with buffer A *plus* 20 mM imidazole, and eluted with buffer A *plus* 300 mM imidazole (direct Ni-NTA). For GRAB, eluted samples were then dialyzed overnight against buffer A *plus* 1 mM 1,4-dithio-DL-threitol (DTT) in the presence of His_6_-tagged TEV at a peptidase:protein weight ratio of 1:100 and 1 mM DTT. The resulting cleavage left additional residues (glycine-alanine-methionine) at the N-terminus of the target proteins due to the cloning strategy (see Table 1). Digested samples were passed several times through Ni-NTA resin previously equilibrated with buffer A *plus* 20 mM imidazole to remove His_6_-tagged molecules and the flow-through containing untagged GRAB was collected (reverse Ni-NTA).

In all cases, proteins eluted from direct and reverse Ni-NTA chromatographies were dialyzed overnight against buffer B (20 mM Tris-HCl, 5 mM sodium chloride, pH 7.5) and further purified by ionic-exchange chromatography (IEC) on a TSKgel DEAE-2SW column (TOSOH Bioscience) equilibrated with buffer B. A gradient of 2–30% buffer C (20 mM Tris-HCl, 1 M sodium chloride, pH 7.5) was applied over 30 mL, and samples were collected and pooled. Subsequently, each pool was concentrated by ultrafiltration and subjected to size-exclusion chromatography (SEC) in Superdex 75 10/300 (GRAB and pro-TGF-β_2_), Superdex 200 10/300 (N-hα_2_M and C-hα_2_M) or Superose 6 10/300 (full-length recombinant hα_2_M) columns (GE Healthcare Life Sciences) in buffer D (20 mM Tris-HCl, 150 mM sodium chloride, pH 7.5). Strep-tagged GRAB was purified by affinity chromatography with Streptactin®XT Superflow Suspension resin (IBA Life Sciences) and eluted with buffer E (100 mM Tris·HCl, 150 mM sodium chloride, pH 8.0) at a further 50 mM in biotin. IEC and SEC purification steps followed as above.

Authentic full-length hα_2_M was isolated from blood plasma from individual donors and purified essentially as described previously^17,24,25^. Briefly, plasma was subjected to sequential precipitation steps with 4–12% PEG 4,000, and the final precipitate containing hα_2_M was reconstituted in 20 mM sodium phosphate at pH 6.4. Partially purified hα_2_M was captured with a zinc-chelating resin (G-Biosciences), washed with buffer F (50 mM sodium phosphate, 250 mM sodium chloride, pH 7.2) *plus* 10 mM imidazole and eluted in the same buffer *plus* 250 mM imidazole and 100 mM EDTA. The protein was first passed through a PD10 desalting column (GE Healthcare Life Sciences) previously equilibrated with 20 mM HEPES, pH 7.5 and then subjected to an IEC step in a Q Sepharose column (2.5 × 10 cm; GE Healthcare Life Sciences), previously equilibrated with 15% buffer G (20 mM HEPES, 1 M sodium chloride, pH 7.5). A gradient of 20–30% buffer G was applied for 150 min and fractions were collected. Collected samples were dialyzed overnight against buffer H (20 mM sodium phosphate, 5 mM sodium chloride, pH 7.4) and further purified by IEC in a TSKgel DEAE-2SW column, previously equilibrated with buffer H. A gradient of 7–20% buffer I (20 mM sodium phosphate, 1 M sodium chloride, pH 7.4) was applied over 30 mL, and samples were collected and pooled. Subsequently, each pool was concentrated and subjected to a final polishing step by SEC in a Superose 6 10/300 column in buffer J (20 mM sodium phosphate, 150 mM sodium chloride, pH 7.4).

Protein identity and purity were assessed by 10–15% Tricine sodium dodecyl sulfate-polyacrylamide gel electrophoresis (SDS-PAGE^26^) stained with Coomassie Brilliant Blue, peptide mass fingerprinting of tryptic protein digests, N-terminal sequencing through Edman degradation, and mass spectrometry. The latter three were carried out at the Protein Chemistry Service and the Proteomics Facilities of the Centro de Investigaciones Biológicas (Madrid, Spain). Ultrafiltration steps were performed with Vivaspin 15 and Vivaspin 500 filter devices of 10- to 50-kDa cut-off (Sartorius Stedim Biotech). Protein concentrations were estimated by measuring the absorbance at 280 nm in a spectrophotometer (NanoDrop) and applying the respective theoretical extinction coefficients. Concentrations were also measured by the BCA Protein Assay Kit (Thermo Scientific) with bovine serum albumin fraction V (BSA; Sigma-Aldrich) as a standard. Induced hα_2_M was obtained by treating native hα_2_M in buffer D with 200 mM methylamine hydrochloride for one hour at room temperature. Subsequently, the sample was dialyzed against the same buffer D.

Human pro-TGF-β_2_ (UP P61812) constructs (see Table 1 and Fig. 1C) were produced in S2 and Expi293F cells and purified as reported elsewhere^8^. Production of mature TGF-β_2_ with a C-terminal His_6_-tag (see Table 1) was assayed with the insect and human systems, which included harvesting periods of seven and three days, respectively. Supernatants were collected after the centrifugation at 2,800 × *g* for 20 min and the purification steps were, first a direct Ni-NTA in buffer A *plus* 20 mM imidazole for the wash step, and *plus* 300 mM imidazole for the elution; and finally purified by SEC with a Superdex 75 10/300 column in buffer D.

### Protein labeling

GRAB and pro-TGF-β_2_ were labelled with fluorogenic sulfosuccinimidyl-7-amino-4-methylcoumarin-3-acetate (Sulfo-NHS-AMCA; Thermo Scientific) according to the manufacturer’s instructions with a 10–15 molar excess of reagent over protein in buffer J for 1 hour at room temperature. Thereafter, the proteins were extensively dialyzed against buffer J to remove non-reacted dye. To assess binding, labelled GRAB or pro-TGF-β_2_ were mixed with authentic hα_2_M (native and induced) or recombinant fragments N-hα_2_M and C-hα_2_M at a 4:1 molar ratio, incubated in buffer J for two hours at 37 °C, and analyzed by 10% native PAGE^27^. Gel fluorescence was visualized in a gel reader (G:BOX F3 Gel Doc System, Syngene) and the fluorescence was measured (*λ*_ex_ = 345–350 nm and *λ*_em_ = 440–460 nm). Negative controls (unlabeled proteins) were included in each experiment. After fluorescence detection, native gels were stained with Coomassie Brilliant Blue (Thermo Scientific) to detect the negative controls.

### Multi-angle laser light scattering

Multi-angle laser light scattering in a Dawn Helios II apparatus (Wyatt Technologies) coupled to a SEC Superose 6 10/300 column (SEC-MALLS) equilibrated in buffer J at 25 °C was performed at the joint IBMB/IRB Crystallography Platform, Barcelona Science Park (Catalonia, Spain) to analyze binding of GRAB or pro-TGF-β_2_ to native or induced authentic hα_2_M at a molar ration of 4:1. ASTRA 7 software (Wyatt Technologies) was used for data processing and analysis, for which a dn/dc value typical for proteins (0.185 mL/g) was assumed. All experiments were performed in triplicate.

### Western blot analyses

Protein samples were separated by 10% SDS-PAGE, transferred to Hybond ECL nitrocellulose membranes (GE Healthcare Life Sciences), and blocked for two hours under gentle stirring at room temperature with 50 mL of blocking solution (phosphate buffered saline; PBS) *plus* 0.1% Tween 20 and 5% BSA. His_6_-tagged proteins were detected by immunoblot analysis using the monoclonal His-HRP Conjugated Antibody (Santa Cruz Biotechnology) diluted 1:5,000 in PBS *plus* 0.1% Tween 20. Strep-tagged proteins were detected with the Streptavidin-Peroxidase Conjugated Antibody from *Streptomyces avidinii* (Sigma-Aldrich) diluted 1:1,000 in PBS *plus* 0.1% Tween 20 and 1% BSA. Complexes were detected using an enhanced chemiluminescence system (Super Signal West Pico Chemiluminescent; Pierce) according to the manufacturer’s instructions. Membranes were exposed to Hyperfilm ECL films (GE Healthcare Life Sciences).

### Proteolytic inhibition assays

Inhibition assays against protein substrates were performed in a microplate fluorimeter (Infinite M200, TECAN) in 200 μL reaction volumes with the fluorescence-based EnzCheck Assay Kit containing BODIPY FL-casein (*λ*_ex_ = 505 nm and *λ*_em_ = 513 nm) as fluorescein conjugate (Invitrogen) at 10 μg/mL in buffer D. Inhibition was measured after preincubation of a two-fold molar excess of authentic or recombinant hα_2_M with trypsin (0.25 μg) for 15 min at room temperature. The substrate was added to the reaction mixture and the residual tryptic activity was measured over a period of two hours.

### Thiol quantification

Detection of free sulfhydryl groups was performed with the Fluorometric Thiol Assay Kit (ab112158 assay; Abcam) following the manufacturer’s instructions and using glutathione as a standard for the dose response curve. The fluorescent signal was measured in a microplate fluorimeter (Infinite M200, TECAN) at *λ*_ex_ = 490 nm and *λ*_em_ = 520 nm in 96-well plates containing 100 μL reaction volumes (50 μL of assay reaction mixture *plus* 50 μL of glutathione-standard or test samples) in duplicate. Fluorescence was measured after preincubation of authentic hα_2_M (0.39 μM) or C-hα_2_M obtained from human cells (1.6 μM), with or without treatment with methylamine for 10, 20, 30, 45 and 60 min, at room temperature.

### Surface plasmon resonance and kinetic data analysis

The binding kinetics (association and dissociation) and affinity (complex formation at the equilibrium) of GRAB or pro-TGF-β_2_ (ligands) with native authentic hα_2_M, induced authentic hα_2_M, recombinant N-hα_2_M or recombinant C-hα_2_M (analytes) were studied by surface plasmon resonance with a Biacore^TM^ T200 Biosensor System (GE Healthcare Life Sciences) at the Scientific and Technological Centers of the University of Barcelona (Catalonia, Spain). To bind ligands provided with a Strep-tag, Streptactin®XT (IBA LifeSciences) was immobilized at 25 °C on the surface of the four flow cells of a sensor chip CM5 series S (GE Healthcare Life Sciences) at 3,000 response units (RU) through amine coupling, as described previously^22^. Subsequently, Strep-tagged GRAB (at 9.7 nM) or pro-TGF-β_2_ (at 19.0 nM) in HBNS buffer (10 mM HEPES, 150 mM sodium chloride, pH 7.4) were immobilized at low RU density on different flow cells of the chip by virtue of the strong interaction between the Strep-tag and streptactin at 5 μL/min for 24 sec at 37 °C. To monitor association, the immobilized ligands were then exposed to the analytes at different concentrations in HBNS (4–600 nM for native and induced authentic hα_2_M; 75–1,200 nM for N-hα_2_M and C-hα_2_M), which were injected at 30 μL/min for 120–240 sec at 37 °C. Thereafter, HBNS was injected for analyte dissociation from the immobilized ligands for 90–300 sec. To dissociate bound ligands and regenerate the chip surface, 3 M guanidine hydrochloride was injected at 30 μL/min for 30 sec after each cycle. These experiments were double referenced by keeping the first flow cell without ligand, and by an injection step at analyte concentration zero. The affinity analysis was performed by plotting binding responses in the steady-state region of the sensorgrams (*R*_*eq*_) against analyte concentrations to determine the overall equilibrium dissociation constant (*K*_*D*_). Sensorgrams were analyzed with the BIAEVALUATION program v. 3.0 (GE Healthcare Life Sciences) and fitted to a 1:1 Langmuir interaction model. The likelihood of fitting was assessed through the *χ*^2^ statistical parameter^28^.

In a separate qualitative experiment, ligands GRAB (at 120 nM) and pro-TGF-β_2_ (at 950 nM) were premixed with the analytes at different concentrations (2–150 nM for native and induced authentic hα_2_M; 38–600 nM for N-hα_2_M and C-hα_2_M) and incubated for one hour at 37 °C. Subsequently, the mixtures were injected at 15 μL/min at 37 °C according to a published multicycle method^29^. The binding was measured through the increase in RU after injection of the premixes and the stability of the resultant complexes through their elution with buffer HBNS at a flow rate of 30 μL/min. Ligand solutions without analyte were used as negative controls of complex formation and the sensor surface was regenerated after each sample injection.

## Results and Discussion

### Biochemical characterization of the recombinant proteins

**Figure 2:**
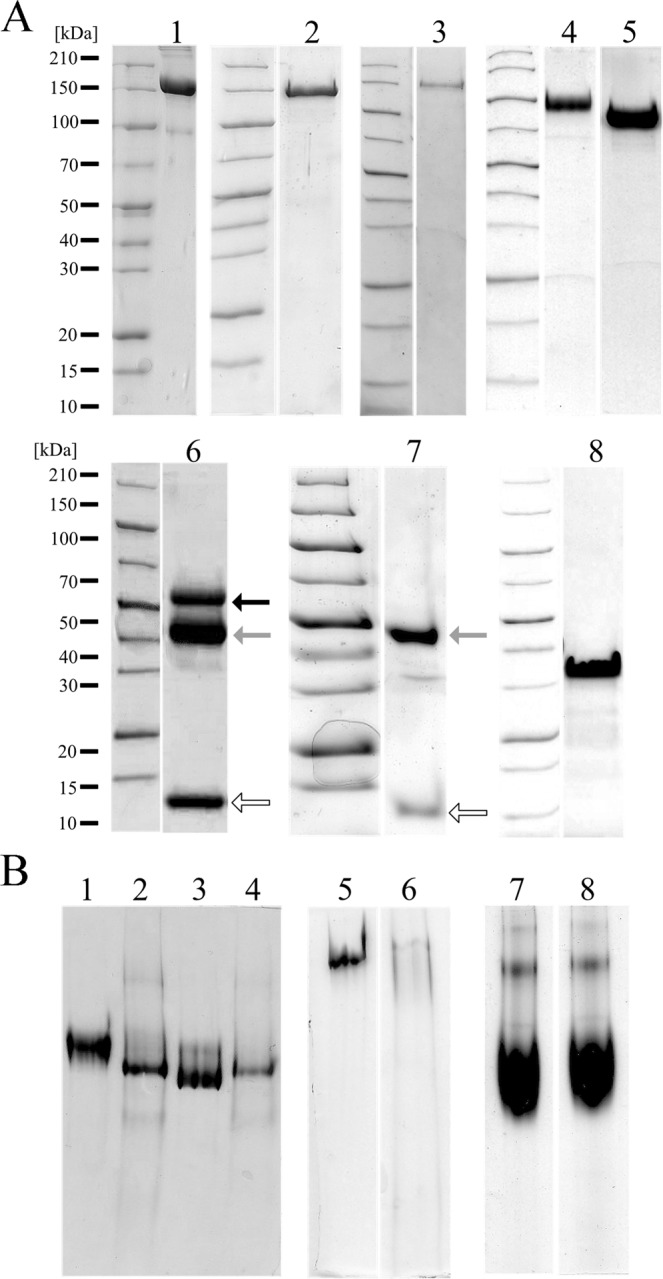
Recombinant protein production and purification. (**A**) SDS-PAGE analysis of wild-type and recombinant proteins. Lanes: 1, native authentic hα_2_M; 2, recombinant hα_2_M from S2 cells; 3, recombinant hα_2_M from Expi293F cells; 4, N-terminal half of hα_2_M (N-hα_2_M); 5, C-terminal half of hα_2_M (C-hα_2_Μ); 6, pro-TGF-β_2_ produced in Expi293F cells according to^8^. Arrows indicate pro-TGF-β_2_ (black), LAP (grey) and mature TGF-β_2_ (white); 7, pro-TGF-β_2_ digested by furin; 8, GRAB. (**B)** Native-PAGE analysis of wild-type and recombinant proteins. Lanes: 1 and 3, native and methylamine-induced authentic hα_2_M; 2 and 4, native and methylamine-induced recombinant hα_2_M from S2 cells; 5 and 6, native authentic hα_2_M and recombinant hα_2_M from Expi293F cells; 7 and 8, native and induced recombinant C-α_2_M expressed from Expi293F cells.

Authentic hα_2_M has been routinely isolated from blood serum, where it is found at an excess of 2–4 mg/mL but is rather heterogeneous as to conformational state, glycosylation and presence of contaminants^17,18^. Native recombinant hα_2_M was obtained from immortalized myelogenous leukemia cell line K-562 but the yield was not reported^14^. Therefore, efforts were made here to develop a system for heterologous expression of the protein with high yield, purity and homogeneity, as well as the necessary flexibility to engineer the protein at will. Full-length hα_2_M with a C-terminal His_6_-tag was expressed in S2 insect cells using a standard transfection protocol^30^ and the signal peptide of the adipokinetic hormone (AKH) for secretion to the extracellular environment. After seven days of expression and harvesting of the supernatant, the protein was purified by affinity chromatography, IEC and SEC steps with yields of up to ~1.0 mg of pure protein per liter of expression medium (Fig. 2A). The protein migrated as a tetramer of ~690 kDa according to SEC (data not shown). Its electrophoretic mobility in native-PAGE was similar to that of induced authentic hα_2_M (Fig. 2B), which migrates faster than the native protein^31^. Chemical treatment with methylamine, which mimics the transition from native to induced hα_2_M by opening the reactive thioester bond to produce a free cysteine^31^, did not have any effect on protein mobility. Consistently, the protein could not inhibit trypsin activity against a fluorogenic protein substrate, even at 10-fold molar excess. We conclude that recombinant hα_2_M produced in insect cells was in the induced form, which does not permit the physiological entrance and entrapment of attacking peptidases^4^, similarly to a previous report of a baculovirus expression system^32^. Moreover, the thioester bond was either not formed or it was opened after secretion into the extracellular environment by nucleophiles from the expression medium. Unfortunately, we could not evaluate this possibility as the composition of the commercial medium that was used is not available. However, the latter hypothesis seems more plausible given that it is reported that insects can produce thioester-containing proteins^33^.

We next developed a transient expression system based on Expi293F cells, which derived from the HEK293 human embryonic kidney cell line and were cultured and harvested at 37 °C for three days. The protein was furnished with the leader sequence of mouse immunoglobulin κ (Ig κ) for secretion and produced ~0.4 mg of pure protein per liter of expression medium (Fig. 2A). The protein migrated as a tetramer in SEC and showed electrophoretic mobility in native-PAGE between native and induced authentic hα_2_M (Fig. 2B). Consistently, its capacity to inhibit trypsin was 35% of native authentic hα_2_M. Together, these data indicate that the recombinant protein is partly native but mainly induced. Previous studies had indicated that thioester formation is a spontaneous process triggered by the packing energy of the polypeptide chain during folding in mammals^34^. Therefore, the limited ability of the Expi293F system to produce native protein was attributed, as in the insect cell system above, to the expression medium rather than to the lack of crucial cell machinery for proper thioester bond formation.

Then we expressed shorter variants of hα_2_M in the insect and mammalian systems (Fig. 2A). N-hα_2_M spanned from macroglobulin-like (MG) domain 1 (MG1) to MG7 in the insect cell system and from MG1 to MG6 in the mammalian system. C-hα_2_M ranged from MG7 to the C-terminal receptor binding domain in both systems (Fig. 1A and Table 1). Expression of N-hα_2_M yielded ~1.6/~3.2 mg per liter of insect and mammalian cell culture, respectively, while the values for C-hα_2_M were ~1.3/~4.6 mg. N-hα_2_M formed a dimer of ~170 kDa due to the presence of an intermolecular disulfide bond (C^278^–C^431^), which is also required for dimerization of the authentic full-length protein (Fig. 1A). Consistently, the protein migrated as a monomer of ~85 kDa in the presence of reducing agents. In turn, C-hα_2_M was monomeric (~75 kDa) and treatment with methylamine did not affect the content of free cysteines or electrophoretic mobility in native-PAGE (Fig. 2B). To follow this up, we qualitatively assayed the content of free sulfhydryl groups by a fluorometric thiol assay kit, which gave a strong fluorescent signal for both the untreated and methylamine-treated C-hα_2_M samples. This contrasted with native full-length authentic hα_2_M, which gave no significant signal, and was similar to methylamine-induced authentic hα_2_M, which likewise gave a strong signal. These assays indicated that the thioester bond was opened in C-hα_2_M as mentioned above for the full-length recombinant variant, possibly owing to a nucleophilic component of the undisclosed cell-growth medium.

The insect and mammalian systems were also assayed for expression of mature human His_6_-tagged TGF-β_2_ (Table 1), but without noticeable yields. Therefore, full-length pro-TGF-β_2_ encompassing LAP and mature TGF-β_2_ (see Fig. 1C) was expressed and purified in Expi293F cells as described elsewhere^8^, with a final yield of ~2.7 mg and ~2.3 mg of N-terminally octahistidine-tagged and Strep-tagged forms, respectively, per liter of mammalian cell culture (Fig. 2A). The protein migrated as a dimer of ~110 kDa in SEC, which indicates that the characteristic disulfide bonds were formed between the LAP and the mature TGF-β_2_ moieties. The purified protein was partially cleaved before residue A^303^ by host peptidases. Subsequent treatment with the physiological activating endopeptidase furin produced a homogenously cleaved species consisting of LAP associated with the mature cytokine (Fig. 2A). Under physiological conditions, TGF-β_2_ maturation is a complex process that involves a cascade of events under participation of several proteins that interact with the initial complex of pro-TGF-β_2_ and the latent TGF-β binding protein (LTBP). LTBP participates as a localizer of pro-TGF-β_2_ to the extracellular matrix, whereas LAP senses the changes and releases mature TGF-β_2_^11^. Previous studies with a Chinese hamster ovary cell expression system benefited from the sensitivity of the LAP domain towards denaturing conditions at very low pH to separate it from mature TGF-β_2_^11,35^. In our case, this was unsuccessful, probably due to different post-translation modifications introduced by Expi293F cells in the highly glycosylated LAP^8,36^.

Finally, full-length GRAB was expressed without the cell-wall anchoring region (Fig. 1B) in a bacterial system yielding ~4 mg of pure protein per liter of expression medium after affinity chromatography, IEC and SEC steps (Fig. 2A). The protein migrated as a ~55-kDa species in SEC and as a ~33-kDa species in SDS-PAGE, but the values determined by SEC-MALLS (15.5 kDa; Table 2) were closer to the theoretical mass (15.8 kDa). We attribute this abnormal migration, which was described previously^11^, to the highly unstructured character of the protein.

**Table 2:** Molecular masses determined by SEC-MALLS.

Protein sample	Molecular mass (kDa)
Native hα_2_M	680.6 ± 1.8
Native hα_2_M + GRAB	707.8 ± 3.4
Induced hα_2_M	684.1 ± 2.7
Induced hα_2_M + GRAB	710.6 ± 1.5
GRAB	15.5 ± 0.0
pro-TGF-β_2_	105.4 ± 0.6

Values are represented as means and standard deviations of three replicates.

### Interaction analysis of hα_2_M and GRAB

**Figure 3:**
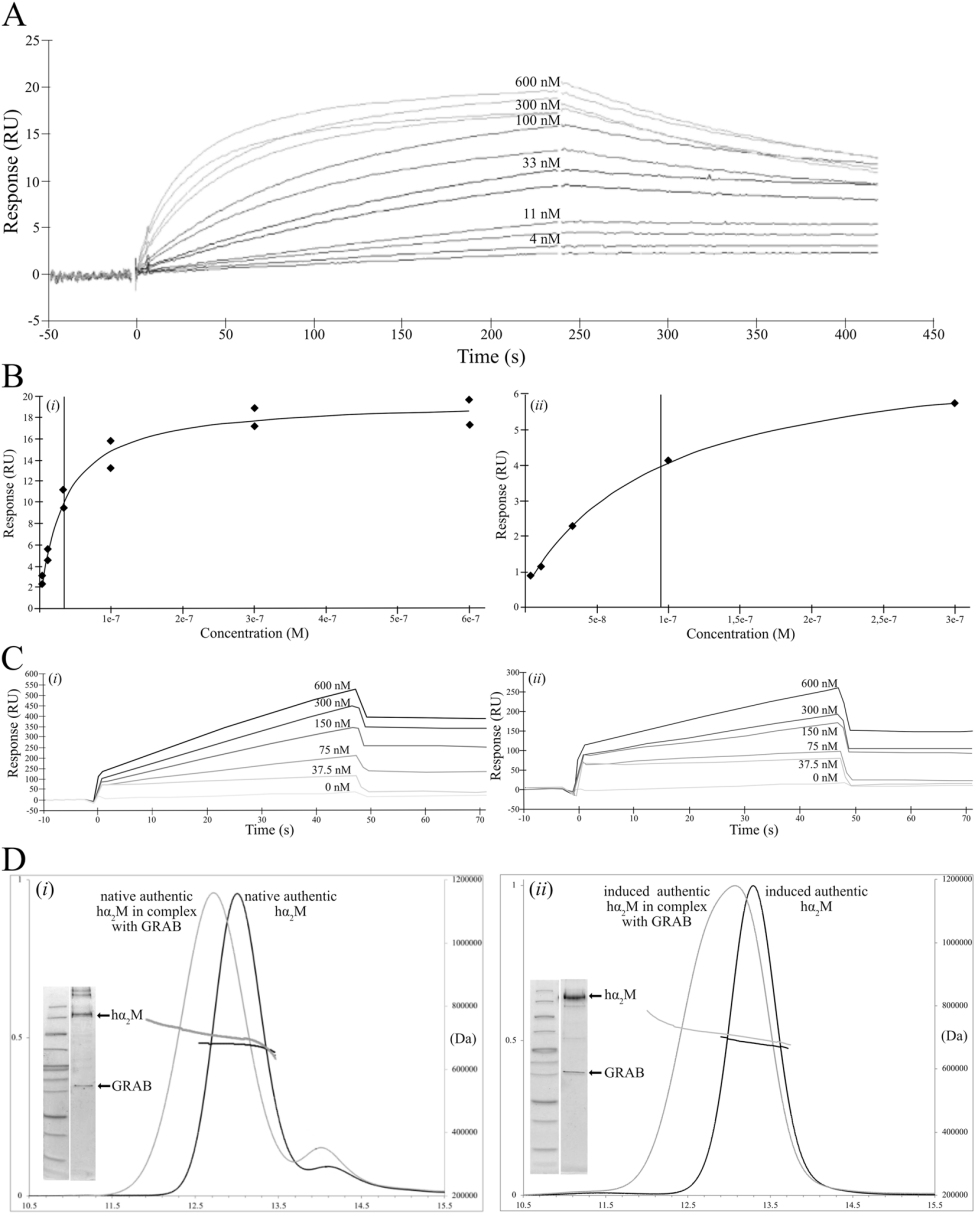
Interaction of GRAB and pro-TGF-β_2_ with hα_2_M variants. (**A**,**B**) Surface-plasmon resonance sensorgrams of the interaction of native or induced authentic hα_2_M with GRAB. Multi-cycle run for native hα_2_M with GRAB (A) and corresponding plot of the steady-state response (B, *i* and *ii*, for native and induced hα_2_M, respectively). Different hα_2_M concentrations were assayed to determine the rate constants that describe the kinetics and the equilibrium constants for complex strength (see also Tables 3 and 4). The vertical line in the plots of steady-state response indicates the value of the calculated equilibrium dissociation constant *K*_D_. (**C)** Sensorgrams of the interaction of N-hα_2_M (*i*) and C-hα_2_M (*ii*) with GRAB. Proteins were premixed, incubated at 37 °C for 1 h, injected over the chip, and the response was measured. **(D)** SEC-MALLS analysis of complex formation between GRAB and native (*left*) and induced (*right*) authentic hα_2_M showing the measured molecular mass distribution. Inserted figures within graphs show the SDS-PAGE analysis of the respective purified complexes.

**Figure 4:**
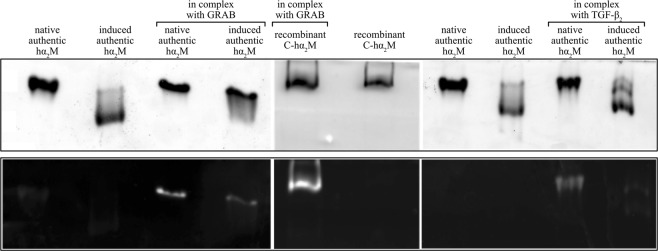
Analysis of complex formation between hα_2_M variants and GRAB or pro-TGF-β_2_. Complexes were separated by native-PAGE. GRAB or TGF-β_2_ labelled with fluorogenic Sulfo-NHS-AMCA were visualized in a gel reader (*lower panels*) and then stained with Coomassie Brilliant Blue (*upper panels*).

Interaction of streptococci with hα_2_M has been reported to be highly specific^15,16^. Group A, G and C streptococci all bind the native form, whereas only the latter interact with the induced form. This result was attributed to the types of surface proteins, which are specific for each strain. GRAB is found in group A streptococci, and we studied its interaction with native authentic hα_2_M by surface plasmon resonance. GRAB was immobilized as a ligand through a Strep-tag on a chip with covalently bound streptavidin. In a multicycle experiment, saturation of the ligand was reached with the two highest analyte concentrations, which gave on- and off-rate kinetic constants and results from affinity analysis (Fig. 3A,B). From the sensorgrams during the sequential injections of different analyte concentrations, we observed fast association and slow dissociation of hα_2_M from GRAB, which indicated stable complex formation. Therefore, the ligand was removed in a regeneration step to make sure that all bound hα_2_M was eliminated between injections with different analyte concentrations. The group of curves in Fig. 3A,B were fitted to a 1:1 Langmuir interaction model. These calculations revealed a *χ*^2^ value < 10% of R_max_, which is indicative of a good fit. Consistently with the sensorgrams, the association rate constant (*k*_a_) and the dissociation rate constant (*k*_d_) were 1.32 × 10^5^ M^−1^s^−1^ and 1.90 × 10^–3^ s^−1^, respectively, with an estimated dissociation halftime (*t*_1/2_ = ln2/*k*_off_) of 365 sec. The equilibrium dissociation constants (*K*_D_) from the kinetic and affinity analysis were 1.43 × 10^−8^ M and 3.45 × 10^−8^ M (Tables 3 and 4), respectively, which indicates high affinity and stable complex formation. The complex was also detected by SDS-PAGE and native-PAGE employing fluorophore-labelled GRAB (Fig. 4). Finally, SEC-MALLS analysis (Fig. 3D and Table 2) showed a molecular mass difference of 27.3 kDa over free hα_2_M, which corresponds to 1.7 molecules of GRAB. Hence, we assume that two molecules of GRAB bind one hα_2_M tetramer.

**Table 3:** Kinetic rates and equilibrium constants of the interaction between native authentic hα_2_M and GRAB.

Protein sample	*k*_a_ (M^−1^ s^−1^)	*k*_d_ (s^−1^)	*K*_D_ (M)	*R*_max_ (RU)	*χ* ^2^
Native hα_2_M + GRAB	1.32 × 10^+5^	1.90 × 10^−3^	1.43 × 10^−8^	18.51	1.23

Constants were calculated from the corresponding plot assuming a 1:1 interaction model (two GRAB molecules per hα_2_M dimer), see Fig. 3A; *k*_a_, association rate constant; *k*_d_, dissociation rate constant; *K*_D_, equilibrium dissociation constant.

**Table 4:** Equilibrium constants of the interaction between native or induced authentic hα_2_M and GRAB.

Protein sample	*K*_D_ (M)	*R*_max_ (RU)	*χ* ^2^
Native hα_2_M + GRAB	3.45 × 10^−8^	18.94	1.17
Induced hα_2_M + GRAB	9.46 × 10^−8^	6.82	0.01

Values were derived from the corresponding plot of steady state response against concentration assuming a 1:1 model (one GRAB molecule per hα_2_M dimer), see Fig. 3B.

Under a similar experimental setup, methylamine-induced authentic hα_2_M was injected over immobilized GRAB to reach equilibrium and saturation, which enabled analysis by affinity. The affinity data permitted calculation with confidence (*χ*^2^ < 10% of R_max_) of the equilibrium dissociation constant (9.46 × 10^−8^ M), which was three times higher than that of native hα_2_M (Fig. 3A,B and Table 4). This is consistent with published results, which indicated that GRAB shows preference for native over protease-induced hα_2_M^16^. The complex was likewise analyzed by SDS-PAGE and native-PAGE with fluorophore-labelled GRAB (Fig. 4). The results showed an increase in the molecular mass of 26.5 kDa over noncomplexed induced hα_2_M, which is equivalent to the results for native hα_2_M.

To map down the region of hα_2_M engaged in GRAB binding, we repeated the above experiments with N-hα_2_M and C-hα_2_M. In a similar multicycle experimental setup, we could not detect any interaction. However, previous incubation of the proteins at 37 °C for one hour apparently enabled complex formation. Protein remained complexed over time after injection and washing of the chip (Fig. 3B), but in this case we could not determine the affinity constants due to the experimental setup. The complexes were subsequently evaluated in native-PAGE using fluorophore-labelled GRAB (Fig. 3D). In this case, we detected the interaction of GRAB with C-hα_2_M but not with N-hα_2_M.

### Interaction analysis of hα_2_M and pro-TGF-β_2_

Previous biochemical data had revealed that hα_2_M binds TGF-β_2_ mainly through a mature cytokine segment spanning residues A^343^-Y^367^, in which W^354^ plays a major role^10^. No data have been reported on the role of LAP. However, inspection of the crystal structure of homologous pro-TGF-β_1_ (see Protein Data Bank code 3RJR^37^) reveals that the interacting segment is partially shielded by LAP. Other studies employing a library of overlapping glutathione *S*-transferase fusion proteins ascribed the potential binding site for TGF-β_1_ to segment V^723^-T^761^ of hα_2_M^38^, which was subsequently narrowed down to E^737^-V^756^ employing synthetic peptides^39^. However, further details on the mechanism are unknown. To further shed light, we set out to characterize binding of pro-TGF-β_2_ to hα_2_M. We checked the interaction by surface plasmon resonance in multicycle experiments with immobilized pro-TGF-β_2_ as ligand but could not detect complex formation. Only after analysis by native-PAGE using fluorophore-labelled pro-TGF-β_2_ we observed interaction with native authentic hα_2_M but not with the induced form or the short variants (Fig. 4). Given that the pro-TGF-β_2_ sample contained a mixture of cleaved and intact protein, we assayed N-terminally His_6_-tagged pro-TGF-β_2_ with native hα_2_M in native-PAGE followed by Western blotting. The two proteins were not co-migrating (data not shown). Thus, we conclude that LAP prevents hα_2_M from binding mature TGF-β_2_ as suggested by structural studies on pro-TGF-β_1_.

### Conclusions

Protein hα_2_M is a sophisticated player to spatially and temporally restrict and regulate key physiological processes that control the distribution and activity of many proteins, including peptidases, cytokines, hormones and other physiological effectors^1^. Since the 1940s, several efforts have been made to understand its mechanism of action *in vivo* and *in vitro*, but they have been hampered by the unavailability of high-yield recombinant expression systems. Here, we developed insect and mammalian systems for the full-length protein and shorter fragments. The former was mainly produced in an induced state, possibly due to media components that cause induction during the time scale of expression. Thus, other media with a regulated composition will be assayed to reevaluate the recombinant systems.

The recombinant proteins *plus* authentic hα_2_M were analyzed for binding with GRAB and pro-TGF-β_2_. The former tightly bound native and methylamine-induced authentic hα_2_M, with two molecules of GRAB per hα_2_M tetramer. The short variants, especially C-hα_2_M, likewise complexed GRAB, but apparently through a different mechanism from the full-length forms. In contrast, full-length pro-TGF-β_2_ did not complex any hα_2_M variant, probably owing to steric hindrance by the N-terminal LAP domain.

## Supplementary information

Original gels and graphs
