# Supplementary material for: Recombinant production of human α2-macroglobulin variants and interaction studies with recombinant G-related α2-macroglobulin binding protein and latent transforming growth factor-β2
Source: Sci Rep. 2019 Jun 24;9:9186. doi: 10.1038/s41598-019-45712-z (PMC6591361; doi:10.1038/s41598-019-45712-z)
Supplement: Supplementary file 1 — Original gels and graphs [file 41598_2019_45712_MOESM1_ESM.pdf]

# Recombinant production of human $\alpha_2$-macroglobulin variants and interaction studies with recombinant G-related $\alpha_2$-macroglobulin binding protein and latent transforming growth factor-$\beta_2$ .

Laura Marino-Puertas[1], Laura del Amo-Maestro[1], Marta Taulés[2], F. Xavier Gomis-Rüth[1,*] and Theodoros Goulas[1,*]

[1]Proteolysis Laboratory; Structural Biology Unit ("Maria de Maeztu" Unit of Excellence); Molecular Biology Institute of Barcelona; Higher Scientific Research Council (CSIC); Barcelona Science Park, Helix Building; Baldiri Reixac, 15-21; 08028 Barcelona (Catalonia, Spain).

[2] Scientific and Technological Centers (CCiTUB); University of Barcelona; Lluís Solé i Sabaris, 1-3; 08028 Barcelona (Catalonia, Spain).

*Corresponding authors: e-mail: xgrcri@ibmb.csic.es and thgcri@ibmb.csic.es.

# Original Gels - Figure 2

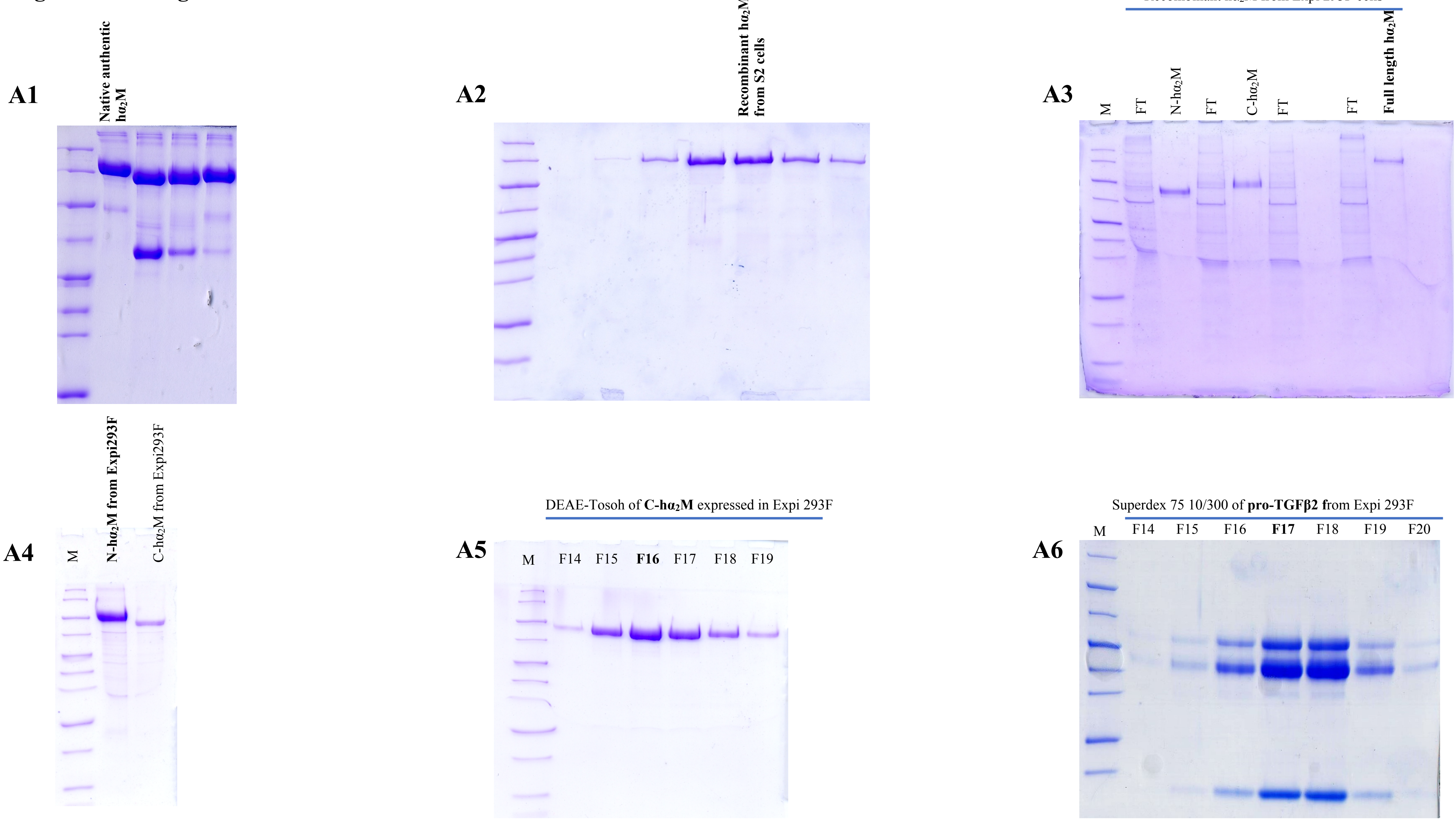

# Original Gels - Figure 2

**A7**

Furin digestion of pro-TGFβ2 expressed in Expi 293F cells

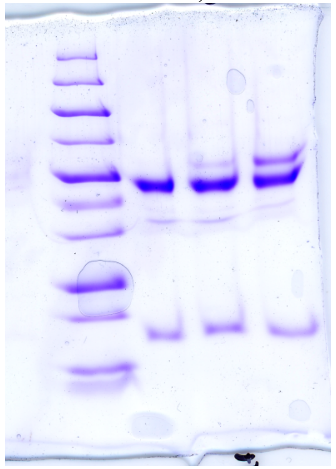


**A8**

Superdex 75 10/300 of **GRAB** expressed in BL21 cells

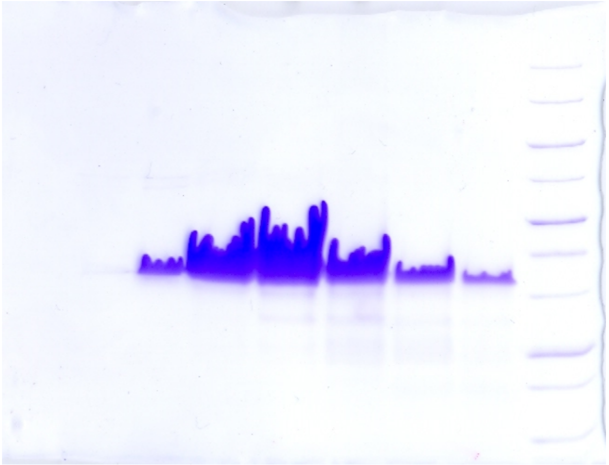


**B1**

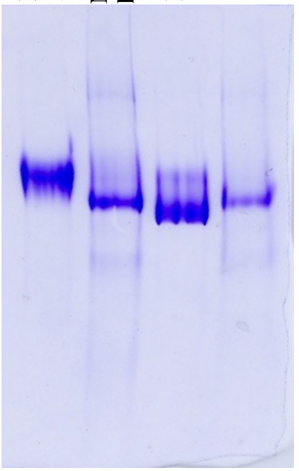


**B2**

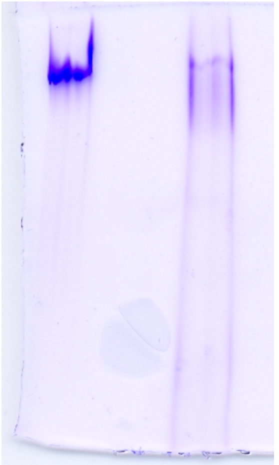


**B3**

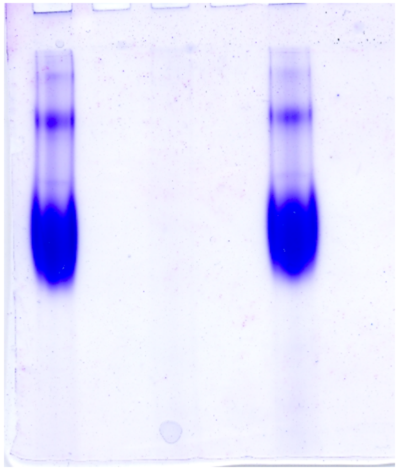

# Original Graphs - Figure 3

A

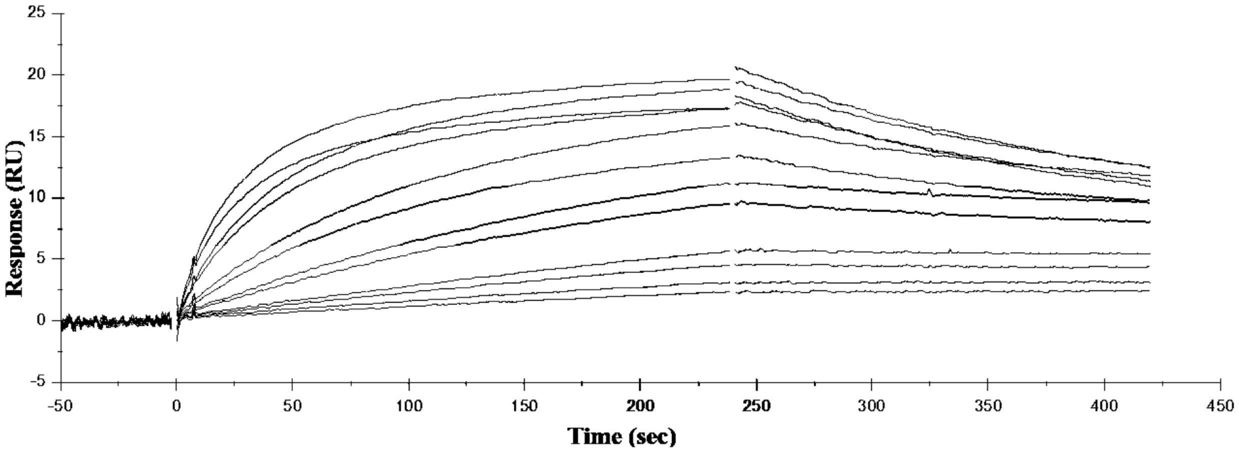


B

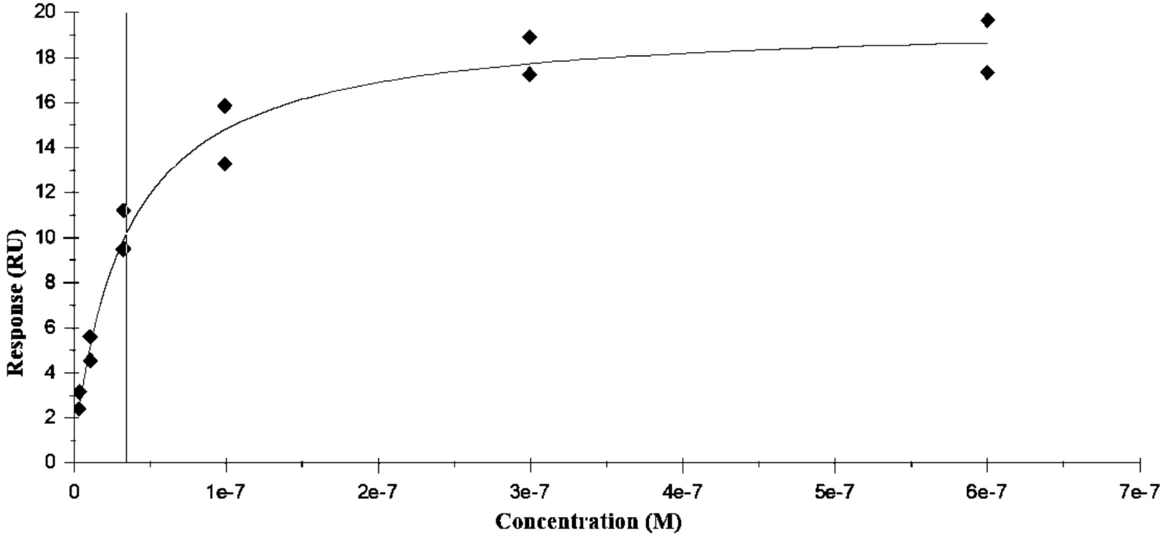


B

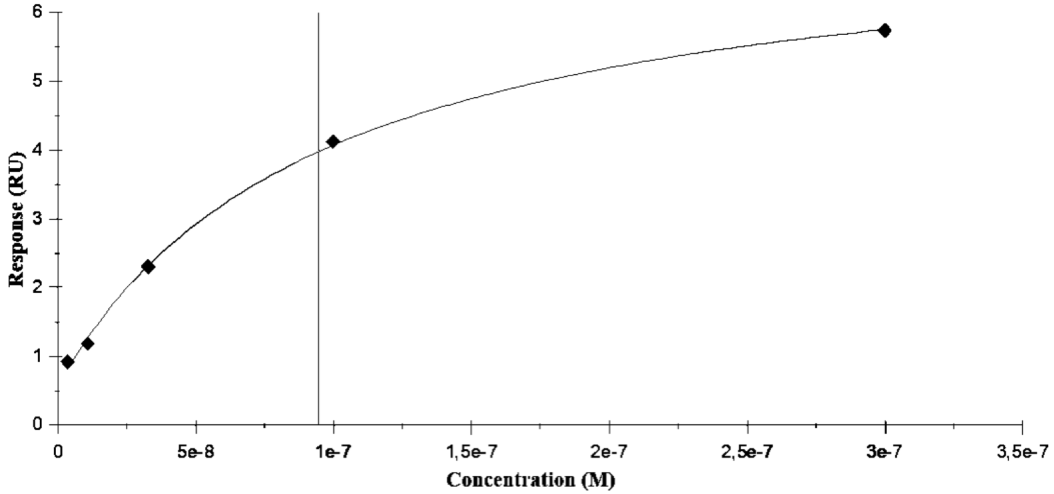

# Original Graphs - Figure 3

**C**

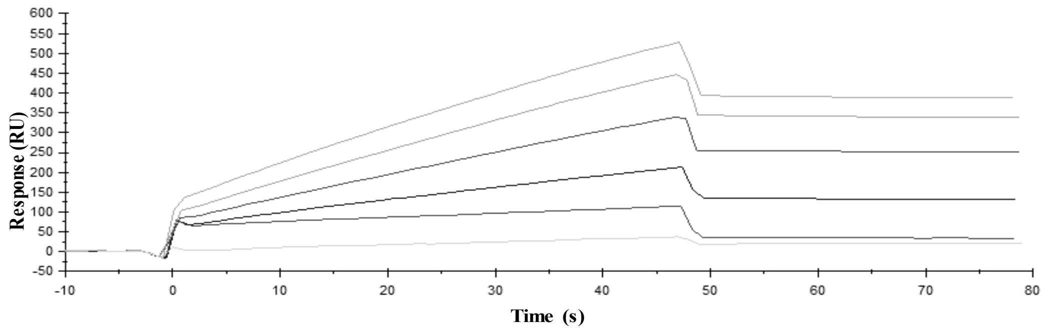


**C**

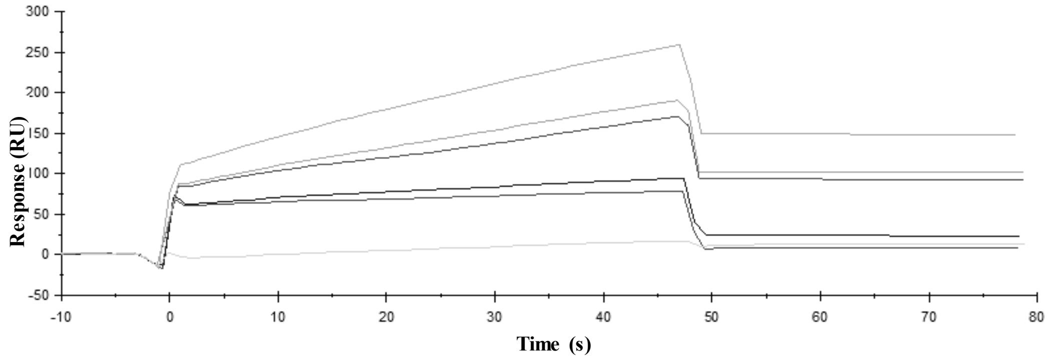

# Original Graphs - Figure 3

**D**

## Native α2M

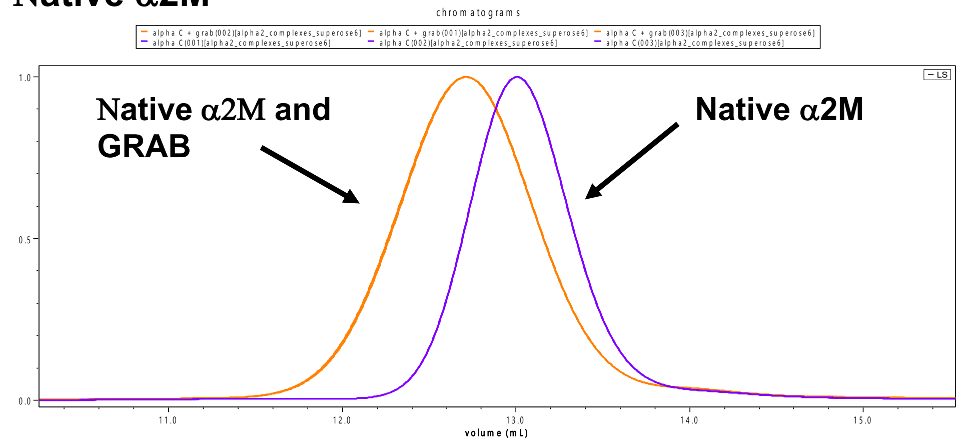


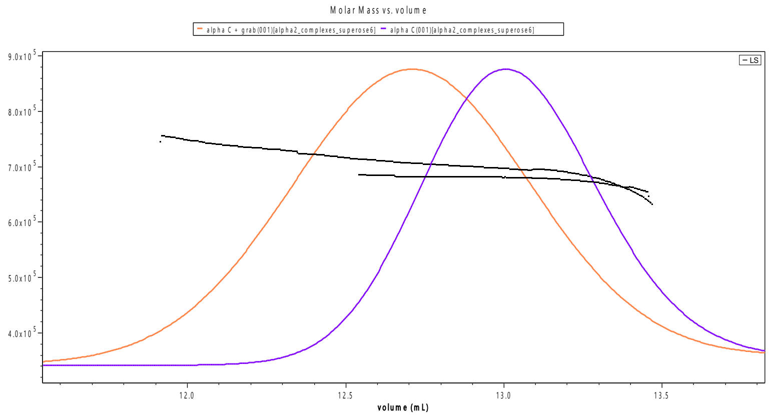


## Induced α2M

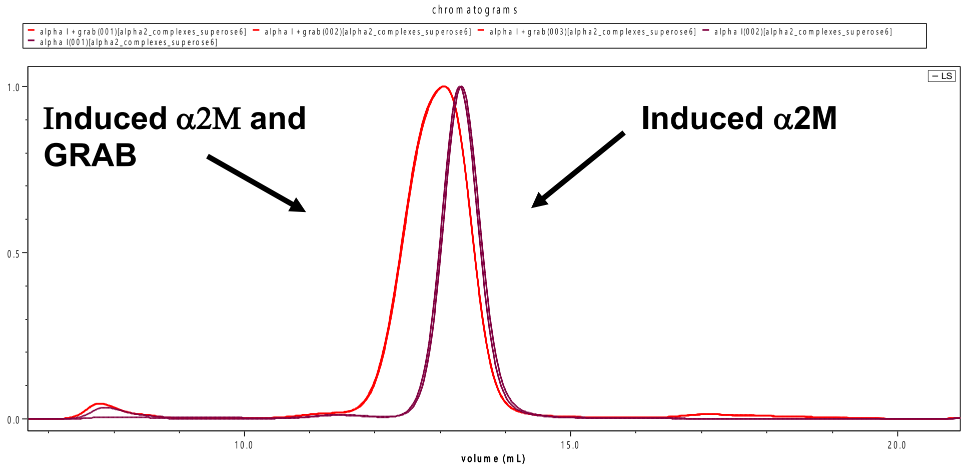


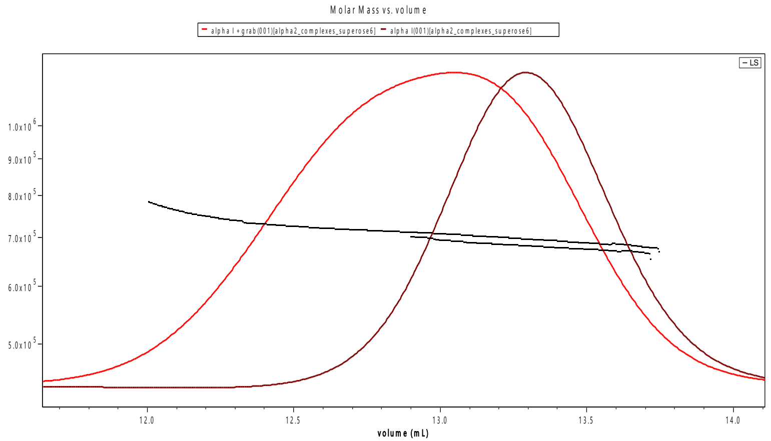

# Original Gels - Figure 4

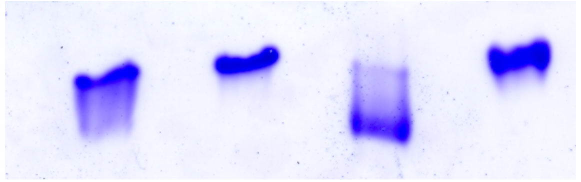


Native gel- Coomassie staining

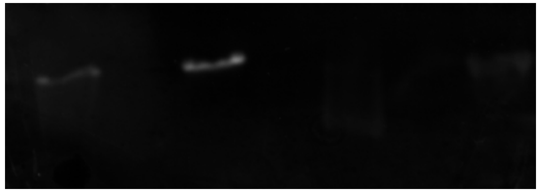


Native gel- Excitation of AMCA at 350nm

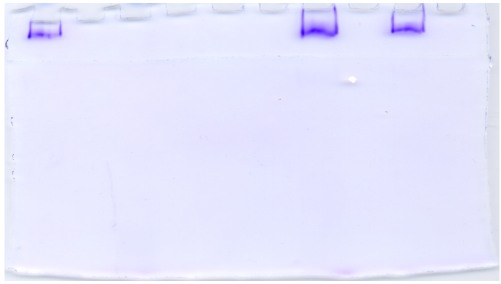


Native gel- Coomassie staining
1,5h running

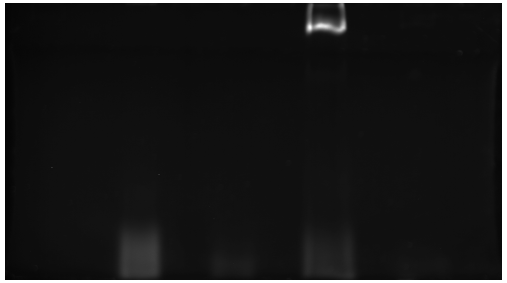


Native gel- Excitation of AMCA at 350nm
1,5h running

# Original Gels - Figure 4

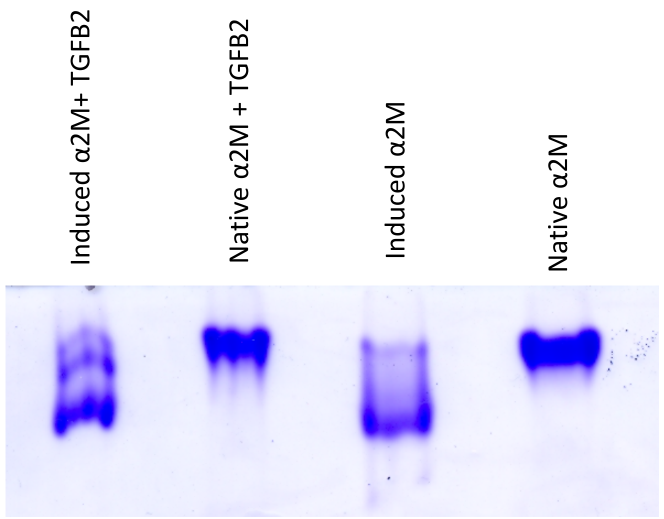


Native gel- Coomassie staining

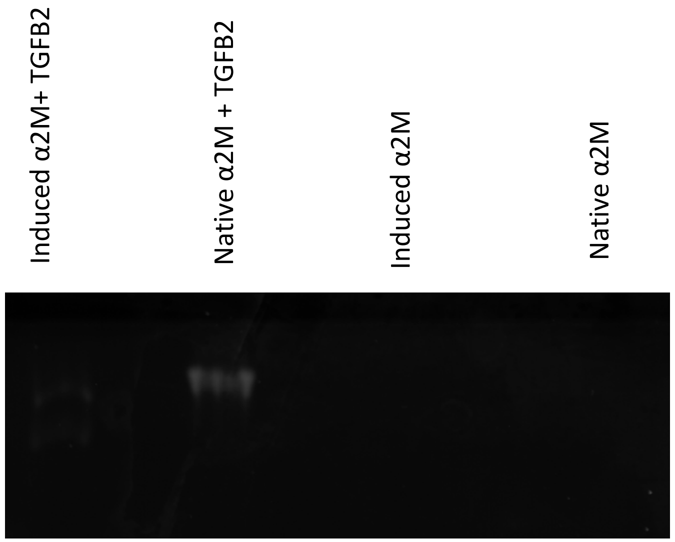


Native gel- Excitation of AMCA at 350nm
